# Functional intron-derived miRNAs and host-gene expression in plants

**DOI:** 10.1186/s13007-018-0351-2

**Published:** 2018-09-24

**Authors:** Umidjon Shapulatov, Mark van Hoogdalem, Marielle Schreuder, Harro Bouwmeester, Ibrokhim Y. Abdurakhmonov, Alexander R. van der Krol

**Affiliations:** 1 Laboratory of Plant Physiology, Wageningen University, Droevendaalsesteeg 1, 6708 PD Wageningen, The Netherlands; 2 Center of Genomics and Bioinformatics, Academy of Sciences of Uzbekistan, University Street-2, Qibray Region, Tashkent, Uzbekistan 111215

**Keywords:** Intron, imiRNA, aimiRNA, miRNA, amiRNA, luciferase

## Abstract

**Background:**

Recently, putative pre-miRNAs locations have been identified in the introns of plant genes, raising the question whether such genes can show a dual functionality by having both correct maturation of the host gene pre-mRNA and maturation of the miRNAs from the intron. Here, we demonstrated that such dual functionality is indeed possible, using as host gene the firefly luciferase gene with intron (ffgLUC), and different artificial intronic miRNAs (aimiRNA) placed within the intron of ffgLUC.

**Results:**

The miRNAs were based on the structure of the natural miR319a. Luciferase (LUC) activity in planta was used to evaluate a correct splicing of the ffgLUC mRNA. Different target sequences were inserted into the aimiRNA to monitor efficiency of silencing of different target mRNAs. After adjusting the insertion cloning strategy, the ffgLUC^aimiR-319a^ gene showed dual functionality with correct splicing of ffgLUC and efficient silencing of TEOSINTE BRANCHED1/CYCLOIDEA/PROLIFERATING CELL FACTOR1 transcription factor genes targeted in-trans by aimiR-319a or targeting the transgene ffLUC in-cis by an aimiR-LUC. Silencing of endogenous target genes by aimiRNA or amiRNA is efficient both in transient assays and stable transformants. A behave as strong phenotype the PHYTOCHROME B (PHYB) gene was also targeted by ffgLUC^aimiR-PHYB^. The lack of silencing of the PHYB target was most likely due to an insensitive target site within the PHYB mRNA which can potentially form a double stranded stem structure.

**Conclusion:**

The combination of an overexpression construct with an artificial intronic microRNA allows for a simultaneous dual function in plants. The concept therefore adds new options to engineering of plant traits that require multiple gene manipulations.

**Electronic supplementary material:**

The online version of this article (10.1186/s13007-018-0351-2) contains supplementary material, which is available to authorized users.

## Background

Important traits of crop plants have successfully been manipulated by selection of mutants [1], by ectopic expression of a transgene [2–4], or by silencing of a single gene [5, 6]. However, because of the complexity of gene-networks in plants, the effect of many single-gene disturbances is limited due to buffering capacity of such networks [7, 8]. Moreover, plant trait manipulation may potentially benefit from synergistic interaction between independent transgene manipulations. Stacking of independent transgenes is time-consuming, especially in crops that are difficult targets for transformation. Engineering in recalcitrant crops may therefore benefit from techniques that can target multiple genes by a single transformation event.

MicroRNAs (miRNA) are short (19-22nt) non coding RNAs that can silence the expression of specific target genes and natural miRNAs form an integral part of developmental decisions in plants [9, 10]. From all plant miRNAs listed in the microRNA database (http://www.mirbase.org/) only a small number have been functionally characterised. Moreover, while most miRNA are processed from regular non-coding miRNA-genes, recently, protein-coding genes with introns containing potential miRNA sequences have been identified both in mammals and plants. For instance, the *Arabidopsis thaliana* genome contains 37 protein coding genes with intronic miRNAs (imiRNAs) and the rice genome contains 181 protein coding genes with imiRNAs [11]. At present, there is no experimental evidence that plant genes containing imiRNAs show simultaneous dual functionality: a correct intron splicing of the host gene pre-mRNA to form a mRNA encoding a functional protein and processing of the miRNA from the intron for effective silencing of the target gene. For instance, in some cases the miRNA encoded in the intron is only produced as alternatively spliced transcript [12]. In such cases, correct mRNA splicing and gene expression and miRNA production from the intron may be mutually exclusive. Functionality of intron-derived miRNAs has been demonstrated in mammals, *C. elegans*, zebra fish, and chicken [13]. It has been demonstrated that an imiRNA can be correctly processed from the intron sequence, without interfering with the accuracy of the splicing process of the host gene [14, 15]. The intron-derived miRNAs require type-II RNA polymerases (Pol-II) and spliceosome components for their biogenesis. In animals, it has been shown that regular miRNA processing is dependent on Drosha-mediated cleavage, but initial processing of some imiRNAs are Drosha-independent. Instead, initial imiRNA processing is coupled to the intron splicing reaction [16]. How imiRNAs are processed in plants is not fully known at present. Introns and active 5′ splice sites (5′ss) have been shown to stimulate the accumulation of miRNAs encoded within the first exons of intron-containing MIR genes and Knop et al. found that the 5′-splice site is crucial for the regulation of intronic miRNA-402 biogenesis from the first intron of host gene At1g77230 [17]. Moreover, the gene encoding dicer protein DCL1 contains imiR838 in intron 14 and the gene can produce both functional DCL1 mRNA and mature miR838. In this instance the imiRNA biogenesis and DCL1 mRNA biogenesis are mutually exclusive but in a population both gene products may be produced [18].

Here, we tested whether a protein coding transgene can be effectively expressed in plants, while also producing a functional miRNA. The feasibility and requirements for such dual gene functionality were determined using three gene construct (ffgLUC^aimiR-319a^, ffgLUC^aimiR-LUC^ and ffgLUC^aimiR-PHYB^), designed to report on both protein and miRNA function. For overexpression, the firefly Luciferase gene with a single intron (ffgLUC) was used, which allows for easy monitoring of gene activity and splicing accuracy. As template for the miRNA sequence in the intron, the sequence of the natural ath-miR319a was used [19]. For alternative targets, the 21-bp sequence targeting TEOSINTE BRANCHED1/CYCLOIDEA/PROLIFERATING CELL FACTOR1 (TCP) transcription factor in miR319a was replaced by a 21 nucleotide sequence targeting ffgLUC mRNA [20] or 21-bp targeting the *Arabidopsis thaliana* PHYB mRNA (AT2G18790). Initially, insertion of the miRNA into the LUC intron resulted in a loss of LUC activity, indicating incorrect splicing of the intron from the LUC pre-mRNA. However, after adjusting the miRNA position within the intron, the transgene showed normal LUC activity when expressed in plants, indicating accurate splicing of the LUC pre-mRNA. Moreover, the aimiRNA targeting TCPs or ffgLUC both were able to suppress target gene expression, indicating effective processing of the aimiRNA from the ffgLUC intron. The concept of a transgene containing an aimiRNA could be useful for simultaneous manipulation of several gene activities, which could be an important tool for plant biotechnology.

## Methods

### Plant materials and growth condition

*Arabidopsis thaliana* (Col-0 background, N1092) was used for stable transformation. The Arabidopsis *phyB*-*9* T-DNA insertion mutant (#CS6217) was obtained from the NASC stock collection. Plants were grown on rock-wool in a growth chamber at 12hL/12hD at 22 °C on half strength Hoagland-nutrient solution.

### Cloning of expression constructs

Artificial microRNAs constructs were created using ath-miR319a backbone as described by Liang [20]. The primer sequences used are listed in Additional file 1: Table S2. The artificial miRNA nucleotide sequences 5′-TAACTGTAAACCGAAAGGCTG-3′ for the *AthPHYB* (AT2G18790) were selected using WMD3-Web MicroRNA Designer (http://wmd3.weigelworld.org/cgi-bin/webapp.cgi). The IDT RNAi design tools (Integrated DNA Technologies) was used to design the amiRNA nucleotide sequence targeting the luciferase mRNA (5′-TAGAACTGCCTGCGTCAGATT-3′). Pre-microRNA 319a was amplified directly from *A. thaliana* genomic DNA using primers (CAAACACACGCTCGGACGCAT-F and CATGGCGATGCCTTAAATAAAG-R). The aimiRNA sequences were amplified from pre-miRNA319a using specific primers which added EcoR V and EcoR I restriction sites for cloning into the intron of ffgLUC (GATATCAGAGAGCTTCCTTGAGTCCATTCAC-F and GAATTCAGGGAGCTCCCTTCAGTCCAATC-R). For amplification of the aimiR-LUC the TCP target sequence in the primers was replaced by the selected LUC target sequence (GATATCTATAACTGCCTGCCTCAGATAAGGTCGTGATATGATTCA-F and GAATTCTAGAACTGCCTGCGTCAGATTAAAGAGAATCAATGATCCA-R). For the amplification of the aimiR-PHYB the TCP target sequence in the primers was replaced by the selected PHYB target sequence (GATATCTAGCTGTAAACCGTAAGGCTCAGGTCGTGATATGATTCA-F and GAATTCTAACTGTAAACCGAAAGGCTGAAAGAGAATCAATGATCCA-R). To generate of ffgLUC _del_^aimiR319a^ construct the first exon plus 10 nucleotides from 5′ site of intron was amplified by using primer which introduce Nco I at start codon and EcoRV site in intron (CCATGGAAGACGCCAAAAAC-F and GATATCAGAAACTTACGTAATGTTCACCTCG-R). The second exon plus 61 base pair from 3′ site of the intron was amplified using primers which introduce an EcoR I site at the end of the intron sequence and an Not I site after the stop codon (GAATTCAACTTTTCTAATATATGACCAAAATTTGTT-F and GCGGCCGCTTACAATTTGGACTTTCCGCCCTT-R). To generate of ffgLUC^imiR319a^ ffgLUC^aimiR-LUC^ and ffgLUC^aimiR-PHYB^ constructs, the first exon plus 33 nucleotides from the 5′ end of the intron was amplified by using primer pairs introducing an Nco I at the ATG start codon and an EcoR V site at the end of the intron sequence (CCATGGAAGACGCCAAAAAC-F and GATATCTACTAATTAATGATAATTATT-R). The second exon of ffgLUC was amplified from 135 base pairs from 3′ splice site to after the stop codon, introducing an EcoR I site at in the intron and Not I site after the stop codon, using the primer pairs (GAATTCGTAATATAATATTTCAAATATTTTTTTCAAAATAA-F and GCGGCCGCTTACAATTTGGACTTTCCGCCCTT-R). The resulting PCR products were digested with EcoR I and EcoR V and the amiRNAs product was ligated into the ffgLUC intron. The ffgLUC was amplified with primers introducing an Nco I site at the ATG and Not I site after the stop codon (CCATGGAAGACGCCAAAAAC-F and CGGCCGCTTACAATTTGGACTTTCCGCCCTT-R). The ffgLUC, ffgLUC^imiR319a^, ffgLUC^aimiR-LUC^ or ffgLUC^aimiR-PHYB^ constructs were subsequently ligated into the Nco I/Not I sites of pIVA2.1 entry vector which contained double 35S promoter and RubescoS terminator. To generate the binary vector, all pIVA2.1-based vectors were cloned into the pKGW_RedSeed vector [21] through gateway based site-specific recombination technology with one way LR reaction. The pKGW RedSeed vector contains a DsRed marker gene that is expressed in the seed coat which allows for selection of T0 transformed seeds.

For confirmation of LUC or PHYB silencing in *trans* the artificial microRNAs 2×35S::amiR-LUC and 2×35S::amiR-PHYB constructs were generated using primer sets which replace the TCP target sequence in miR-319a with target sequences for LUC or PHYB respectively (LUC: CCATGGTATAACTGCCTGCCTCAGATAAGGTCGTGATATGATTCA-F and GCGGCCGCTAGAACTGCCTGCGTCAGATTAAAGAGAATCAATGATCCA-R or PHYB: CCATGGTAGCTGTAAACCGTAAGGCTCAGGTCGTGATATGATTCA-F and GCGGCCGCTAACTGTAAACCGAAAGGCTGAAAGAGAATCAATGATCCA-R). The PCR products were cloned into pIVA2.1 entry vector which was subsequently used for recombination into the pKGW_RedSeed vector. All destination vectors were subsequently transformed to *Agrobacterium tumefaciens* (AGL0).

### Plant transformation and selection transformants

*Agrobacterium tumefaciens* was used for plant transformation using the floral dip method as described [22]. Transgenic T_0_ seeds were identified by DsRed pigmentation of the seed coat. For germination seeds were plated on 3% water agar plates and cold-treated for 5 days at 4 °C after which plates were incubated in growth chambers in the light at room temperature. After 3 days, germinated seedlings were transferred to soil or rock wool for plant growth.

### *Agrobacterium*-*mediated transient expression in N.benthamiana* leaves

Agro-infiltration in *N. benthamiana* using agrobacterium strains carrying the different expression vectors (or empty vector) was done as described by Wang [23].

### *LUC* activity measurement

For LUC activity measurements in stable transformed *Arabidopsis thaliana* plants were sprayed with 1 mM D-luciferin (Duchefa, Haarlem, NL) 24 h and 1 h before imaging with an (− 80 °C) air-cooled CCD Pixis 1024B camera system (Princeton Instruments, Massachusetts, USA) equipped with a 35 mm, 1:1.4 Nikkon SLR camera lens (Nikon, Tokyo, Japan) fitted with a DT Green filter ring (Image Optics Components Ltd, Orsay, France) to block chlorophyll fluorescence. Exposure time is as indicated.

For transient assays, *N.benthamiana* leaves were harvested 4 days post agro-infiltration. Leaves were sprayed with 1 mM D-luciferin at 24 and 1 h before imaging (5 min exposure time). Relative luminescence from LUC activity was analysed in Image J (Bethesda, Maryland, USA), using background subtraction. For each treatment LUC activity in leaves from 6 to 8 independent plants was quantified.

### Hypocotyl length measurement

For hypocotyl length measurement, seeds were surface sterilized and imbibed on 0.25% water agar plates at 4 °C in the dark, after which plates were transferred to a Red LED light box (50 uMol) at 22 °C. Seedlings were flattened at 5 days after transfer, and hypocotyl length was determined from photograph in Image J (Bethesda, Maryland, USA). At least 20 seedlings were scored from each genotype.

### Quantitative RT-PCR

For RNA analysis, T_3_ generation plants were grown for 4 weeks. The RNA was extracted from rosette leaves from WT (Col-0), ffgLUC_del_^aimiR-319a^, ffgLUC^aimiR-319a^ or ffgLUC^aimiR-PHYB^ transformants using InviTrap Spin Plant RNA mini Kit (Berlin, Germany), following manufacturer’s instructions. Purified total RNAs were subjected to TURBO DNA-free™ DNase (Thermo Fisher Scientific Inc., Waltham, Massachusetts) treatment to avoid with contaminated genomic DNA. For reverse transcription the iScrip II mix reagent was used that included 10 mM oligo (dT) primer according to the manufacturer’s instruction (Bio-Rad, CA,USA). The primers listed in Additional file 1: Table S2 were used for the real time qPCR. Reaction were carried out with RNA isolated from pooled samples from three individual plants, with triple biological replicates using SYBR Green PCR Master Mix (Bio-Rad, CA,USA) on the CFX Connect Real Time System machine (Bio-Rad, CA, USA). For Arabidopsis the *A. thaliana ACTIN1* was used as reference. RNA analysis from transient assays in *N. benthamiana* were carried out on RNA isolated from three pooled agro-infiltrated leaves, in triple biological replicates, using *N.benthamiana UBI3* as reference genes. The Ct method (2 − ΔΔCt) was used to analysis the differences in mRNA values (http://www.bio-rad.com/). All expression constructs used in the transient assays contain a 35S::DsRed marker gene and quantification of the DsRED gene expression in the transient assays was used to confirm similar transformation frequencies in the different agro-infiltration treatments.

### Small RNA extraction and stem-loop RT-PCR assays

Detection of specific small RNAs was by the step-loop PCR method as described by Varkonyi-Gasic [24]. Briefly, leaf material was collected from ffgLUC (as control), ffgLUC_del_^aimiR-319a^, ffgLUC^aimiR-319a^, and ffgLUC^aimiR-PHYB^ plants and immediately ground in liquid nitrogen with a mortar and pestle. Approximately 100 mg ground leaf tissue was used to small RNA extraction. The extraction of small RNAs were performed by using Prima microRNA Isolation Kit (Lot#SLBL6958 V, Sigma Aldrich, USA) according to the manufacturer’s protocol. The small RNA purity and concentration was measured by NanoDrop spectrophotometer (Thermo Scientific, USA).

The specific RT primers were used for miR319a and amiR-PHYB in stem-loop RT reaction. Reverse transcription reaction were performed according to Varkonyi-Gasic et al. [24]. Forward primers for mature miR319a or amiR-PHYB and universal reverse primer (see Additional file 1: Table S2) were used in RT-PCR. The PCR amplification products analysed by gel-electrophoresis on a 4% agarose gel in 1xTAE buffer.

### Statistical analyses

Comparison of means was analysed for statistical significance with a 2-sample *t* test (*P* < 0.001).

## Results

### ffgLUC gene with intron-deletion miR-319a (ffgLUC_del_^aimiR-319a^) shows only single functionality: impaired LUC mRNA splicing but efficient silencing of TCP targets

**Fig. 1:**
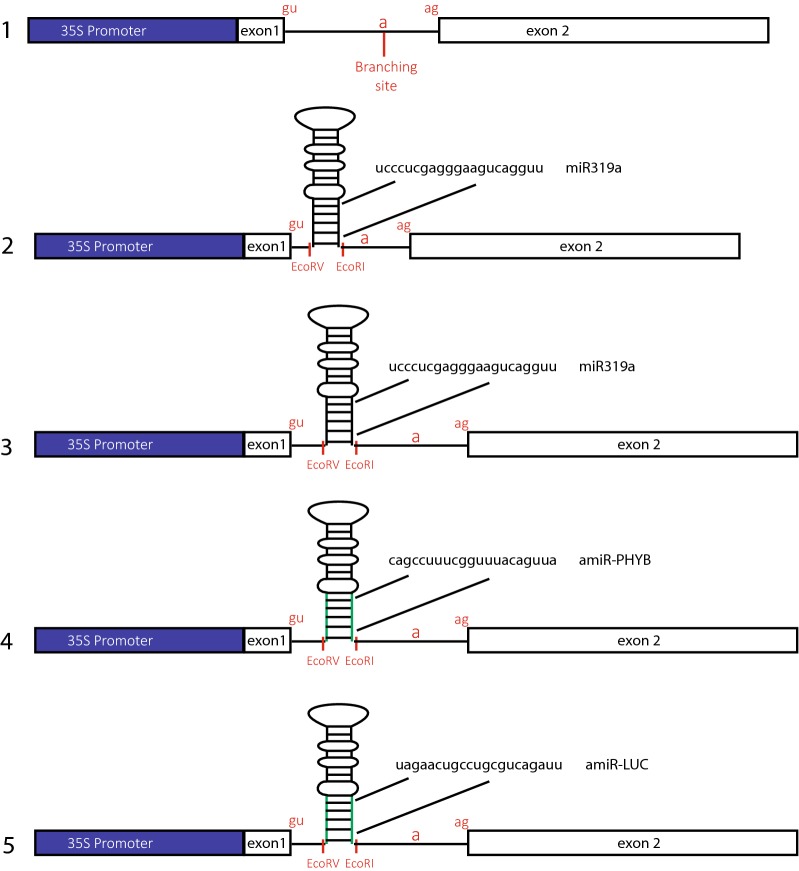
Structure of aimiRNA expression constructs. **1** The Firefly Luciferase gene with intron 2×35S:ffgLUC. **2** aimiRNA gene with miR319a in ffgLUC intron with small deletion: 2×35S:ffgLUC_del_^aimiR-319a^. **3** aimiRNA gene with miR319a in ffgLUC intron without deletion: 2×35S:ffgLUC^aimiR-319a^. **4** aimiRNA gene targeting *AthPHYB*: 2×35S:ffgLUC^aimiR-PHYB^. **5** aimiRNA gene targeting ffLUC: 2×35S:ffgLUC^aimiR-LUC^. “*a*” indicates the intron branch point site, “*gu*” indicates the 5′-splice site and “*ag*” indicates the 3′-splice site. Exchange of 21 bp target sequence in miR319a for LUC or PHYB target sequences is indicated with green lines

To determine whether a functional microRNA can be efficiently generated from an intron of a transgene, without affecting accuracy of intron splicing, both accuracy and efficiency of transgene splicing and efficiency of silencing by the aimiRNA need to be monitored. To monitor transgene splicing the firefly luciferase (ffgLUC) reporter gene with an intron was used [25] (see Fig. 1). To study the efficiency of target gene silencing, the precursor of the native miRNA319a, which targets several members of the *A*rabidopsis TCP transcription factor family, was used [26]. When the artificial intron-miRNA, aimiR-319a, is correctly processed, it should be active and elicit a leaf growth phenotype similar to that induced by 2×35S::miR-319a [20].

**Fig. 2:**
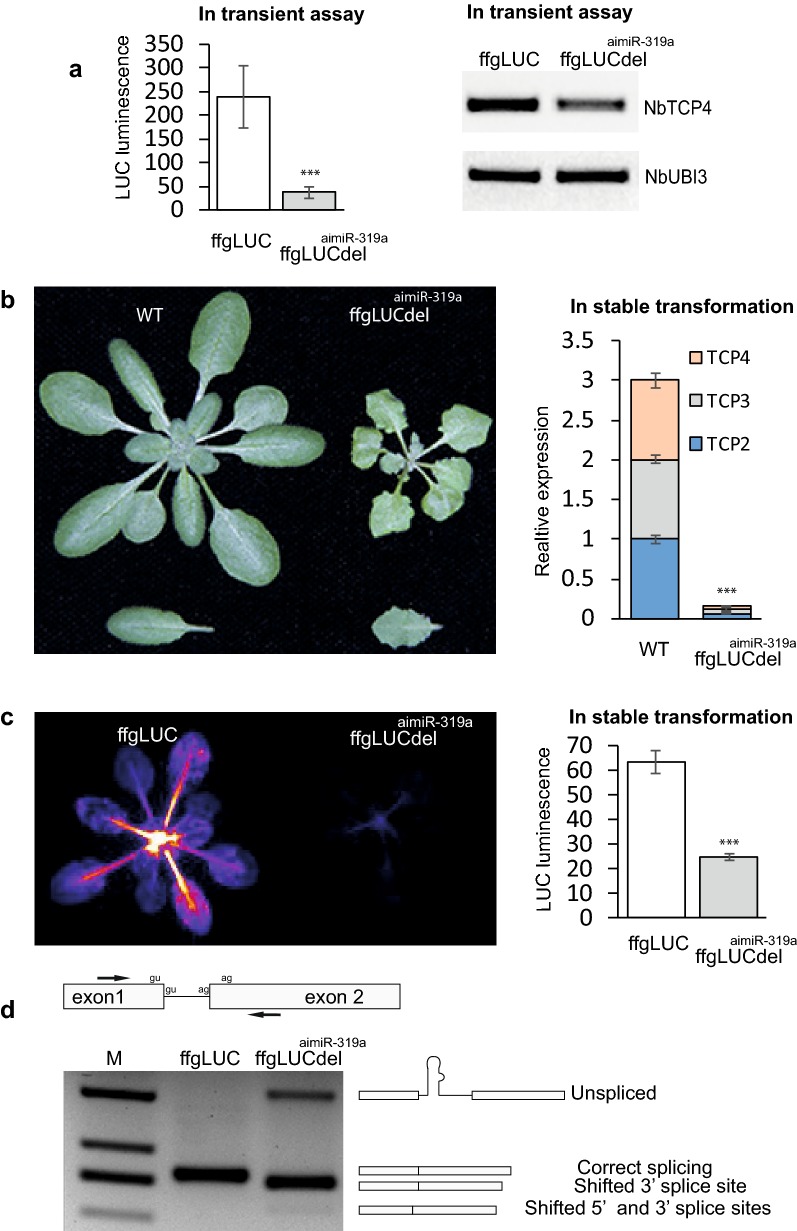
Single activity of ffgLUC_del_^aimiR-319a^ in transient assays and stable transformed plants. **a** Left: LUC activity of ffgLUC and ffgLUC_del_^aimiR-319a^ at 4 days post-agro-infiltration in *Nicotiana benthamiana* transient assay. Significant differences between samples (***) is based on standard error (student’s *t* test, *P* < 0.01). Right: RT-PCR analysis of *NbTCP4* expression in transient assay with ffgLUC or ffgLUC_del_^aimiR-319a^. Quantification of the DsRED gene expression was used to confirm similar transformation efficiencies in the agro-infiltration with ffgLUC and ffgLUC_del_^aimiR-319a^ (Additional file 1: Fig. S3). **b** Left: Representative stable transformed Arabidopsis rosette plant and leaf expressing ff-gLUC or ffgLUC_del_^aimiR-319a^. Right: (reference gene *AthActin1*, expression of *TCP* 2, 3 and 4 each normalized to that in one WT plant). Significant differences between samples (***) is based on standard error (student’s *t* test, *P* < 0.01). **c** LUC activity in representative stable transformant Arabidopsis expressing ff-gLUC or ffgLUC_del_^aimiR-319a^. Graph: quantified LUC expression of eight independent transformants expressing either ff-gLUC or ffgLUC_del_^aimiR-319a^. **d** Top: PCR forward and reverse primer positions in ffgLUC_del_^aimiR-319a^. Bottom: PCR products on RNA isolated from ffgLUC_del_^aimiR-319a^ plants. At each band position the structure of the mRNA sequence is shown (see also Additional file 1: Fig. S2)

The initial cloning procedure for insertion of the miR-319a precursor sequence into the intron of ffgLUC resulted in a 37 base pair deletion in the ffgLUC intron (for sequence see Additional file 1: Fig. S1). This gene is named ffgLUC_del_^aimiR-319a^ (see Fig. 1, 2). In ffgLUC_del_^aimiR-319a^ the intron branch point and both 5′ and 3′ intron border sequences remained intact (see Additional file 1: Fig. S1). The ffgLUC_del_^aimiR-319a^ was cloned into a binary expression vector under control of the enhanced CaMV 2×35S promoter and a red seed coat transformation marker gene [21]. The ffgLUC_del_^aimiR-319a^ expression construct was introduced into *Agrobacterium tumefaciens* and activity of the constructs was tested both by transient expression in *N.benthamiana* leaves and by stable transformation of *A.thaliana*. In the transient expression assay in *N. benthamiana*, LUC activity of ffgLUC_del_^aimiR-319a^ was compared to that of a ffgLUC at 4 days post-agro infiltration. Results show a high LUC activity in leaves expressing ffgLUC, but only low LUC activity for leaves expressing ffgLUC_del_^aimiR-319a^ (Fig. 2a). This indicates that intron splicing accuracy from ffgLUC_del_^aimiR-319a^ is impaired compared to that of ffgLUC. The transient expression assays in *N. benthamiana* are not suitable to assess if aimiR-319a elicits a leaf phenotype. Therefore, we tested whether endogenous *N. benthamiana TCP4* (*NbTCP4*) gene expression was affected by ffgLUC_del_^aimiR-319a^ as the *AthTCP* target sequence of aimiR-319a shows substantial overlap with sequences in *NbTCP4*. Results show that *NbTCP4* expression level was reduced in leaves expressing ffgLUC_del_^aimiR-319a^ compared to the control leaves expressing ffgLUC (Fig. 2a).

Transformants of *Arabidopsis thaliana* with the ffgLUC_del_^aimiR-319a^ or ffgLUC expression constructs were identified in T_0_ seeds by expression of the red seed coat marker present in the binary vector (Additional file 1: Table S1). From the red ffgLUC_del_^aimiR-319a^ T_0_ seeds 19 independent transformants were grown. All these plants showed a leaf growth phenotype (data not showed) as described for plants expressing 2×35S::miR319a [20], indicating that the miR319a is efficiently processed from aimiR-319a in stable transformed plants. Indeed, expression analysis of the miR319a target genes *AthTCP2*, *AthTCP3*, *AthTCP4* indicated that their expression was reduced by ~ 90% in the ffgLUC_del_^aimiR-319a^ plants (Fig. 2b). However, the LUC activity in plants expressing ffgLUC_del_^aimiR-319a^ is low compared to control plants (expressing ffgLUC) of the same age (Fig. 2c). Both the reduced LUC activity of ffgLUC_del_^aimiR-319a^ in transient assay and stable transformants suggest an incorrect maturation of the luciferase pre-mRNA derived from the ffgLUC_del_^aimiR-319a^. Indeed, PCR analysis of the luciferase mRNA across the intron splice site showed that there were multiple aberrant products and only very low levels of correctly spiced luciferase mRNA derived from ffgLUC_del_^aimiR-319a^ (Fig. 2d). Presumably, the dual action at the intron in luciferase pre-mRNA by both an intron-splicing protein-complex and an miRNA processing protein-complex leads to spatial interference, which in this case especially affects correct maturation of the pre-mRNA. Sequence analysis of the aberrant PCR products showed that both aberrant 3′- and 5′ splice site selection occurred, while the major PCR product was derived from unspliced mRNA (Fig. 2d and Additional file 1: Fig. S2). To solve the putative spatial interference during processing of ffgLUC_del_^aimiR-319a^ mRNA maturation, we next adapted the cloning strategy for miRNA insertion into the intron.

### ffgLUC^aimiR-319a^ displays dual functionality: correct LUC mRNA splicing and TCP silencing

**Fig. 3:**
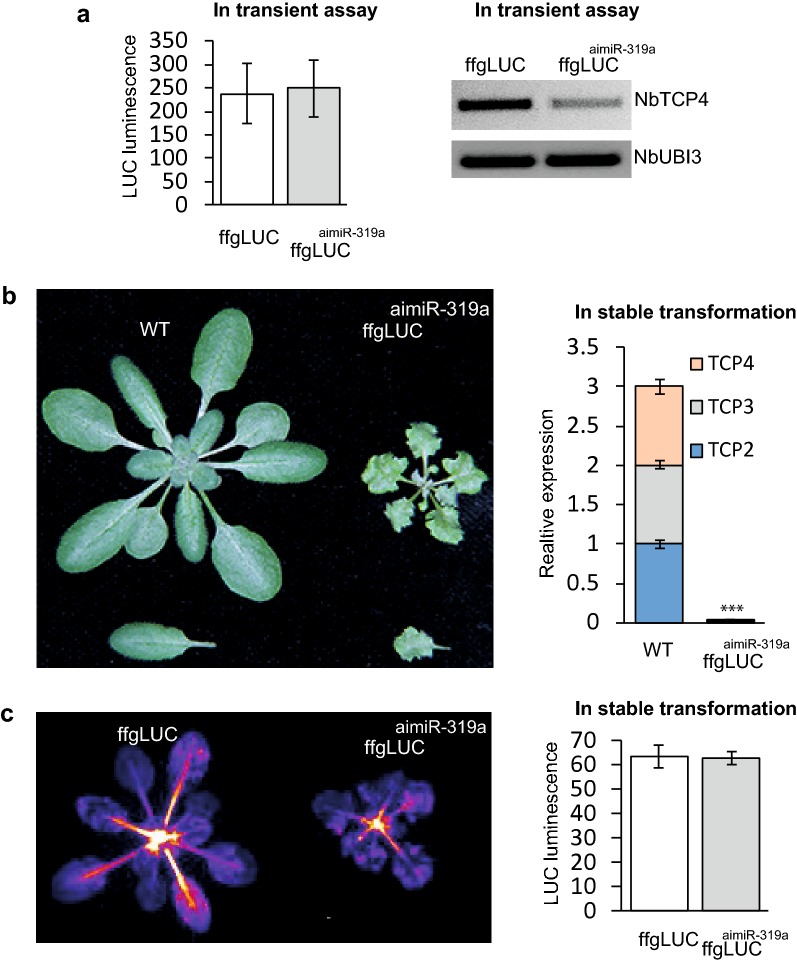
Dual activity of ffgLUC^aimiR-319a^ in transient assays (**a**) and stable transformed plants (**b**, **c**).** a** Left: LUC activity of ffgLUC and ffgLUC^aimiR-319a^ at 4 days post-agro-infiltration in *Nicotiana benthamiana* transient assay (n = five leaves per treatment). Right: RT-PCR analysis of *Nb*TCP4 expression in transient assay with ffgLUC or ffgLUC_del_^aimiR-319a^. Quantification of the DsRED gene expression was used to confirm similar transformation efficiencies in the agro-infiltration with ffgLUC and ffgLUC^aimiR-319a^ (Additional file 1: Fig. S3). **b** Left: Representative stable transformed *Arabidopsis thaliana* rosette plant and leaf expressing ff-gLUC or ffgLUC^aimiR-319a^ Right: average relative expression level of *TCP*2/3/4 genes in five WT and ffgLUC_del_^aimiR-319a^ plants (reference gene *AthActin1*, expression of *TCP*2,3 and 4 each normalized to that in one WT plant). Significant differences between samples (***) is based on standard error (student’s *t* test, *P* < 0.01). **c** Left: LUC activity in representative stable transformant *Arabidopsis thaliana* expressing ff-gLUC or ffgLUC^aimiR-319a^. Right: quantified LUC in expression of eight independent transformants expressing either ff-gLUC or ffgLUC^aimiR-319a^

The miRNA insertion cloning strategy was adapted by direct insertion of the aimiRNA into the ffgLUC intron, without deletion of intron sequence, resulting in the expression construct ffgLUC^aimiR-319a^ (Fig. 1 and Additional file 1: Fig. S1). The ffgLUC^aimiR-319a^ expression construct was introduced into *Agrobacterium tumefaciens* and was again tested both by transient expression in *N. benthamiana* and by stable transformation of *Arabidopsis*. In the transient expression assays, the activity of ffgLUC^aimiR-319a^ was compared with that of ffgLUC without intronic miR319a (Fig. 3a). This resulted in a similar LUC activity in leaf tissue expressing either ffgLUC^aimiR-319a^ or ffgLUC, suggesting an efficient and accurate splicing of the intron from ffgLUC^aimiR-319a^ mRNA (Fig. 3a). To test the functionality of aimiR-319a in targeting TCP genes in *N. benthamiana*, *NbTCP4* mRNA level was checked by RT-PCR in control treatments and leaves expressing ffgLUC^maimiR-319a^. *NbTCP4* expression was reduced by 60% in leaves infiltrated with ffgLUC^aimiRNA319a^, suggesting that a functional miRNA319a can be produced from aimiR-319a (Fig. 3a).

From the stable transformation of *Arabidopsis*, T_0_ seeds expressing the red seed coat marker were selected, from which 19 independent T1 transformants were grown (Additional file 1: Table S1). Out of these 19 plants, two plants did not survive, while 17 plants produced T_1_ seeds. Each of these 17 T_1_ plants showed the phenotype associated with constitutively overexpressed native miR319a [20, 27] (Fig. 3b). This indicates that an miR319a was efficiently processed from imiR-319a located in the intron of ffgLUC^aimiR-319a^ in stably transformed plants, leading to efficient silencing of TCP genes. This is also confirmed by qPCR analysis of RNA isolated from a representative ffgLUC^aimiR-319a^ transformant, which shows > 90% reduction in *TCP2, TCP3* and *TCP4* mRNA levels compared to plants expressing conventional ffgLUC (Fig. 3b). Nevertheless, LUC activity in the same ffgLUC^aimiR-319a^ transformant is similar compared to the ffgLUC control (Fig. 3c). These results indicate that the luciferase pre-mRNA is correctly spliced and simultaneously aimiR-319a provides silencing of TCPs in transformed plants.

### aimiR-LUC silences ffgLUC in-*cis* in stable transformants, but not in transient assays

**Fig. 4:**
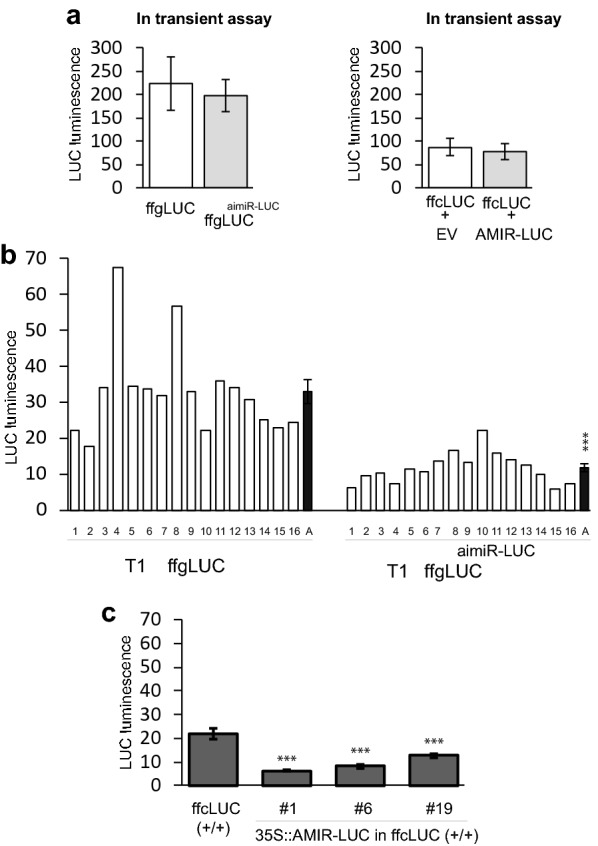
Evaluation of silencing in *cis*- and *trans* in transient assays (**a**) and stable transformed plants (**b**, **c**).** a** Left: evaluation silencing in-cis transient assay: LUC activity of ffgLUC and ffgLUC^aimiR-LUC^ at 4 days post-agro-infiltration in *Nicotiana benthamiana* transient assay (n = five leaves per treatment). Right: evaluation silencing in-trans transient assay: LUC activity in *Nicotiana benthamiana* transient assay of ffcLUC co-infiltrated with empty vector and ffcLUC co-infiltrated with amiR-LUC at 4 days post-agro-infiltration (n = five leaves per treatment). Quantification of the DsRED gene expression was used to confirm similar transformation efficiencies in the agro-infiltration with ffgLUC and ffgLUC^aimiR-LUC^ (Additional file 1: Fig. S3). **b** Evaluation of silencing in-cis: Relative LUC activity in sixteen independent T_1_ generation of ffgLUC and ffgLUC^aimiR-LUC^ plants. “A” indicates average LUC activity in set of transgenic plants. Significant differences between samples (***) is based on standard error (student’s *t* test, *P* < 0.01). **c** Evaluation of silencing in-trans: Relative LUC activity in homozygous ffcLUC line and T1 of same ffcLUC line transformed with 2×35S:amiR-LUC. Significant differences between samples (***) is based on standard error (student’s *t* test, *P* < 0.01)

An aimiRNA was made targeting the luciferase mRNA itself (aimiR-LUC). The aimiR-LUC is based on the sequence and structure of the native miR319a precursor, but the 21 base-pair sequences targeting TCPs are replaced by 21 base-pairs targeting luciferase mRNA (Fig. 1 and Additional file 1: Fig. S1). In cells expressing ffgLUC^aimiR-LUC^, aimiR-LUC targets expression of the LUC transgene from which it is derived (silencing in-*cis*). Both mature aimiR-LUC and LUC mRNA are produced from the same pre-mRNA and silencing of LUC activity provides information on the relative efficiency of the two maturation processes (mRNA vs miRNA). In the transient expression assays, LUC activity in *N. benthamiana* leaves expressing ffgLUC^aimiR-LUC^ showed no significant reduction compared with leaves expressing ffgLUC (Fig. 4a). This indicates correct splicing of the luciferase pre-mRNA, but no effective silencing by aimiR-LUC in-*cis*. To compare the silencing in-*cis* with silencing in-*trans* in the transient assay, an ffcLUC (LUC cDNA) expression construct was co-infiltrated with a 2×35S::amiR-LUC expression construct. This showed that also 2×35S::amiR-LUC is not capable of silencing transiently expressed LUC (Fig. 4a). Combined, these results indicate that efficient maturation of luciferase mRNA from ffgLUC^aimiR-LUC^ occurs upon transient expression but that silencing by aimiR-LUC or amiR-LUC is not effective under these conditions.

The ffgLUC and ffgLUC^aimiR-LUC^ binary vectors were also stably transformed into *Arabidopsis* and T_0_ seeds with the red seed coat were identified (Additional file 1: Table S1). For each transformation event, 16 independent transformants were grown and LUC activity was quantified in independent transformed plants at 21 days post germination. On average, the LUC activity was reduced by 65% in the 16 individual ffgLUC^aimiR-LUC^ T_1_ plants compared to that in 16 individual T_1_ ffgLUC plants (Fig. 4b). This indicates that amiR-LUC is efficiently processed from aimiR-LUC in stably transformed plants. For comparison of silencing in-*cis* with silencing in-*trans* in stably transformed plants, one line expressing 2×35S::ffcLUC was transformed with a 2×35S::amiR-LUC expression construct. In three T2 double transformants (homozygous for both 2×35S::ffcLUC and 2×35S::amiR-LUC) the LUC activity was reduced by 69–53% compared to the original ffcLUC line (Fig. 4c). Silencing efficiency in-*trans* therefore seems to be in the same range as silencing efficiency in-*cis*.

### ffgLUC^aimiR-PHYB^ shows efficient mRNA maturation, but no silencing of *AthPHYB*

**Fig. 5:**
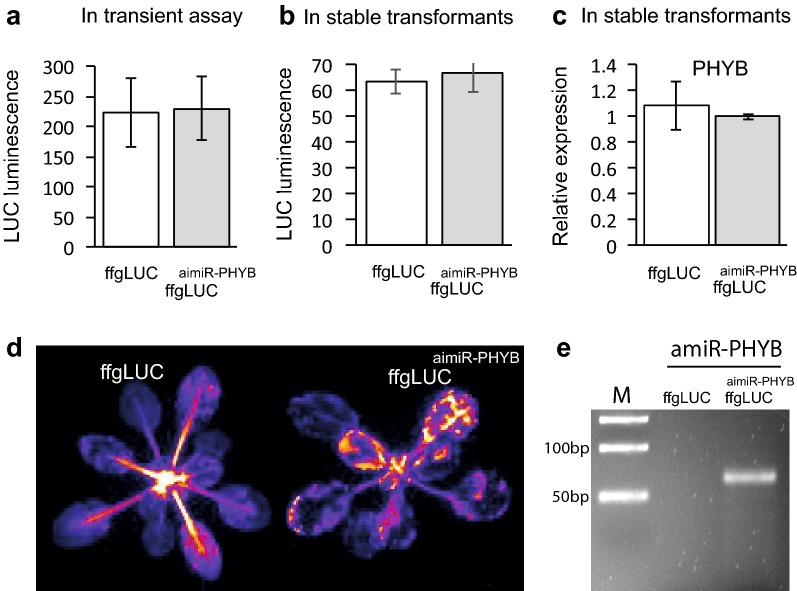
Activity of ffgLUC^aimiR-PHYB^ in transient assays (**a**) and stable transformed plants (**b**, **c**). **a** Relative LUC activity of ffgLUC and ffgLUC^aimiR-PHYB^ at 4 days post-agro-infiltration of *Nicotiana benthamiana* leaves (n = five leaves per treatment). **b** Relative LUC activity in eight independentstable transformants of *Arabidopsis thaliana* expressing either ff-gLUC or ffgLUC^aimiR-PHYB^. **c** Average relative expression level of *AthPHYB* in five ff-gLUC and ffgLUC^aimiR-PHYB^ plants (reference gene *AthActin1*, expression of *AthPHYB* normalized to that in one ffgLUC plant). **d** Image of LUC activity in representative transgenic plants expressing ff-gLUC (left) or ffgLUC^aimiR-PHYB^ (right). **e** Detection of mature amiR-PHYB by stem-loop RT PCR analysis in small RNA isolated from ffgLUC^aimiR-PHYB^ plants but not in small RNA isolated from control ffgLUC plants

In addition to the aimiRNA targeting TCP and LUC, an aimiRNA targeted against the *PHYB* mRNA of *Arabidopsis thaliana* was tested. The aimiR-PHYB was again placed at the same intron position as in the functional ffgLUC^imiR-319a^ and ffgLUC^aimiR-LUC^ constructs. The aimiR-LUC is based again on the miR319a but with a replacement of the 21 base pairs in miR319a targeting TCP by 21 base pairs targeting *AthPHYB* mRNA (ffgLUC^aimiR-PHYB^) (Fig. 1 and Additional file 1: Fig. S1). In transient assays, the leaf tissues expressing ffgLUC^aimiR-PHYB^ showed similar LUC activity as leaves expressing ffgLUC (Fig. 5a), again indicating efficient and accurate maturation of the luciferase pre-mRNA from the ffgLUC^aimiR-PHYB^ expression construct.

After stable transformation of *Arabidopsis thaliana* with ffgLUC^aimiR-PHYB^, T_0_ seeds with the red seed coat were identified (Table 1) and 10 independent transformed T_1_ plants were grown. From these, eight transformants with a single copy transgene insertion were selected for further analysis. On average, the LUC activity in these eight lines was comparable with that of eight independent transformants expressing ffgLUC (Fig. 5b). This again indicates that also the intron in ffgLUC^aimiR-PHYB^ is efficiently and correctly spliced from the luciferase pre-mRNA. In contrast, the silencing of PHYB is not effective in ffgLUC^aimiR-PHYB^ lines as expression of PHYB mRNA is similar in ffgLUC^aimiR-PHYB^ and ffgLUC lines (Fig. 5c). The silencing of PHYB mRNA expression can also be tested in a bioassay. When seedlings are grown under constant Red (cR) light, lines with reduced PHYB expression are expected so show enhanced hypocotyl elongation. Ten independent homozygous T1 lines of ffgLUC^aimiR-PHYB^ were germinated under cR. While the hypocotyl length of a *phyB*-*9* mutant was elongated compared with WT, the hypocotyl length of the ten ffgLUC^aimiR-PHYB^ transformants was not statistically different from WT (data not shown). All together, these results suggest that the mature amiR-PHYB derived from ffgLUC^aimiR-PHYB^ is not functional in silencing PHYB expression.

## Discussion

### Functional aimiRNA requires sufficient spacing in intron

Intron-derived miRNAs (imiRNAs) are an alternative source for miRNAs in mammals and plants [16, 28–31]. Evidence has been obtained that functional miRNAs can be derived from imiRNAs in mammalian cells and plants [32, 33] but plant genes containing intronic miRNA sequences have only been studied sparsely. Here, we demonstrate that the concept of an imiRNA can be used to construct a transgene with dual functionality: overexpression of the transgene and silencing of an endogenous target gene of interest. Our constructs demonstrate that the structural sequence information of the pre-miRNA mi319a is sufficient for full functionality when placed correctly into an intron, allowing for both normal maturation of the pre-mRNA and for generation of a functional mature microRNA.

In all aimiRNA constructs tested here, the aimiRNA was inserted into an 189 long intron sequence of the ffgLUC gene. In the first construct the insertion was done at 10 bp from the 5′-end of the LUC-intron sequence. For this construct the LUC activity was low compared to ffgLUC control construct in both in transient and stable (Fig. 2a, c). This indicates that a certain distance is needed between the 5′-splice site and the imiRNA insertion site for efficient pre-mRNA maturation. It could be that the reduced distance between 5′-splice site and imiRNA sequence in ffgLUC_del_^aimiR-319a^ resulted in spatial constraints because of simultaneous assembly of spliceosome and miRNA-processing protein complexes. In contrast, the aimiRNA placed at 55 bp from the 5′-splice site resulted in efficient maturation of the LUC mRNA, resulting in similar LUC activity for ffgLUC^aimiRNA-319a^ and ffgLUC in transient expression as well as stable transformants (Fig. 3a, c). It was not investigated whether mRNA and aimiRNA derive from the same pre-mRNA transcript or whether the two mature products are produced mutually exclusive. However, since LUC activity from ffgLUC^aimiRNA-319a^ is similar as from ffgLUC it suggests the same level of mRNA production from both constructs. If part of the pre-mRNA is exclusively used for mature amiRNA production and the other part for mature ffgLUC mRNA production we would expect a lower LUC activity from ffgLUC^aimiRNA-319a^, which is not the case (Fig. 3). Whether both products (mRNA, aimiRNA) are indeed derived from the same pre-mRNA needs further investigation but for practical purposes the ffgLUC^aimiRNA^ constructs seem to function as dual functional transgenes.

The imiRNA positioning within the intron may be further improved for functionality, for which positioning of natural imiRNA in plant genes may be used as a guide. The average length of introns is 101 bp in *Arabidopsis* and 160 bp in rice [34, 35]. By contrast, the average length of introns containing imiRNAs is 625 bp in *Arabidopsis* and 2178 bp in rice [11]. Therefore, it may still be possible that a larger distance between inserted miRNA and the 5′- and 3′ splice sites enhances functionality of the imiRNA (more efficient splicing and processing to miRNA).

### Efficiency of silencing is function of both aimiRNA and target gene expression level

The construct ffgLUC^aimiRNA-LUC^ with the miRNA targeting the LUC mRNA in-*cis* showed ~ 65% reduction in LUC activity, which is very similar to the silencing in-*trans* reached by a 2×35S::amiR-LUC in stably transformed plants (Fig. 4b, c). Presumably the LUC mRNA and aimiR-LUC are produced in equal molar amounts from ffgLUC^aimiRNA-LUC^ pre-mRNA, suggesting that miRNA needs to be in excess to its target mRNA in order to obtain higher levels of silencing. For instance, silencing of the *TCP* transcription factor genes, which are expressed at low levels, by the aimiRNA is very efficient (Fig. 2b, 3b). In contrast to the stable transformed plants, the constructs targeting LUC mRNA in-*cis* or in-*trans* are not effective in transient assays. In transient assays the gene copy number is artificially high and may result in saturation of the gene silencing machinery.

### amiRNA-PHYB not functional because of target mRNA secondary structure?

**Fig. 6:**
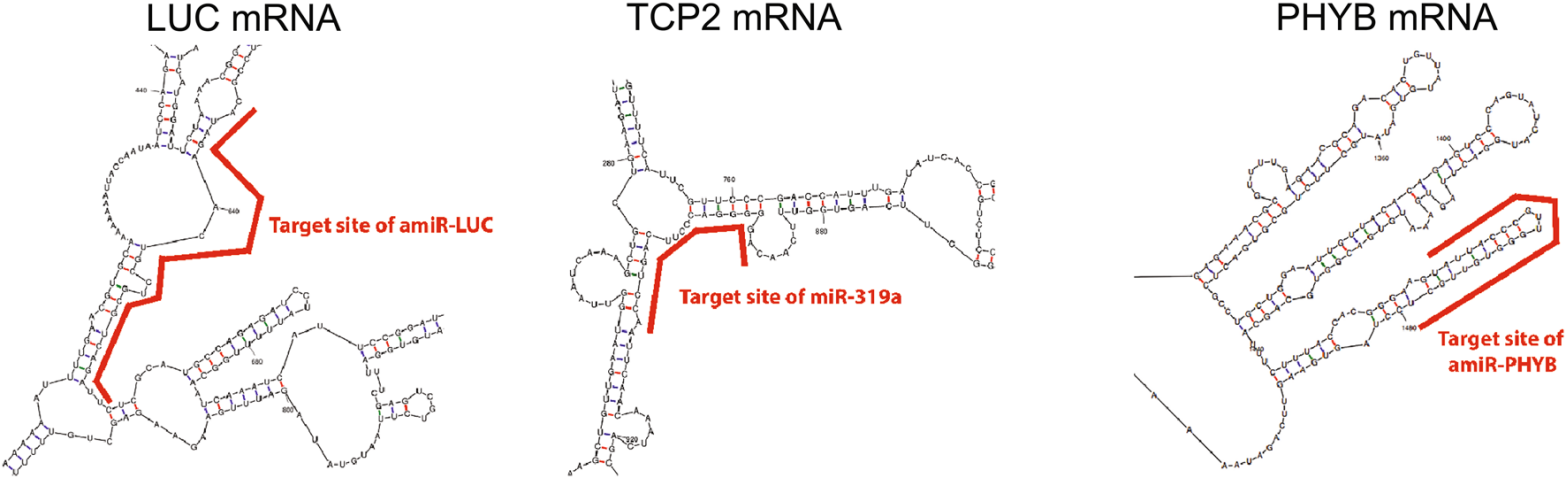
Predicted secondary structure of mRNAs targeted by miRNA. RNA secondary structure prediction by UNAFold (http://unafold.rna.albany.edu/) [37]

The construct ffgLUC^aimiRNA-PHYB^ showed correct splicing but this did not result in significant down regulation of *PHYB* mRNA levels in transformed *Arabidopsis*. Analysis of small RNA isolated from the plants expressing ffgLUC^aimiRNA-PHYB^ by stem-loop PCR [24] with specific primers did show that the expected aimiRNA^PHYB^ product is produced in these plants (Fig. 5e), but apparently it is not active against the PHYB mRNA. Also when the same amiR-PHYB was expressed directly from a 2×35S-promoter, transformants did not show a PHYB silencing phenotype under constant cR (Additional file 1: Fig. S5). The lack of silencing by either aimiR-PHYB or amiR-PHYB suggests that the PHYB target sequence cannot be effectively silenced. For selection of the PHYB miRNA target sequence the WMD3-Web MicroRNA Designer online tool was used, which selects the best target sequence based on both target and off-target sequences [5]. However, recently it was shown that effectiveness of miRNA sequences also depends on the secondary structure of the target mRNA [36]. Indeed, when the secondary structure of the target mRNA is taken into account [37], it turns out that both the amiR-LUC and native miR319a target the mRNA at a part that is largely single stranded. In contrast, the chosen amiR-PHYB sequence targets the *PHYB* mRNA at an internal stem loop structure (Fig. 6). Possibly, this explains why the ffgLUC^aimiR-PHYB^ construct does not show effective silencing of *PHYB*.

In conclusion, the method with transgenes containing an amiRNA in their intron allows for combining ectopic overexpression of the transgene with silencing of a target gene of interest. Artificial miRNA genes containing functional clusters of miRNAs have been engineered [38]. Therefore, our concept of transgenes containing aimiRNA may be extended by multiple aimiRNAs in a single intron or in different introns in the same transgene provided that these aimiRNAs are still efficiently processed and allow dual/multiple functionality of the transgene.

## Additional file

**Additional file 1: Figure S1.** Nucleotide sequences of all ff-gLUC/aimiRNA constructs used in this study. gatatc: − EcoR V site; gaattc: EcoR I site; capital letter: exon sequences; small letters black: intron sequences; small letters blue: Id-amiRNA sequences based on miR319a. Specific sequences targeting TCP, LUC or PHYB are underlined. 5′intron splice sequences are boxed (ag: 3′intron splice site, gt: 5′intron splice site). **Figure S2.** RT-PCR products sequence from ffgLUC_del_^aimiR-319a^ transgenic plant. **Figure S3.** A similar transformation efficiency were confirmed by quantify the DsRED gene expression in transient assay samples. The quantification data is normalized against the *N. benthamiana* reference with UBI3 as internal control. Error bars represent standard error. **Figure S4.** Confirmation of mature aimiR-319a expression in stem-loop RT-PCR assay. Illustration of RT-PCR method for amplification of mature microRNA (**A**). Gel electrophoresis results from stem-loop RT (**B**). ffgLUC plant used as a positive control for endogenous mature miR319a. A mature microRNA specific forward primer and universal reverse primer were used for PCR amplification. Mature microRNA products were obtained using 25 cycling of RT-PCR and analysed on 4% agarose gel in 1xTAE. Predicted products were compared with 50 bp DNA ladder. **Figure S5.** PHYB silencing phenotype under constant cR. **Table S1.** Stable transformation of Arabidopsis WT or Arabidopsis line ff-gLUC-1 with the different expression constructs. *NA* not applicable, *very low LUC activity; **low LUC activity. **Table S2.** List of primers were used in this work.

## Abbreviations

imiRNA: Intronic microRNA,
aimiRNA: Artificial intronic microRNA
ffgLUC: Firefly genomic luciferase
TCP: TEOSINTE BRANCHED1/CYCLOIDEA/PROLIFERATING CELL FACTOR1
PHYB: PHYTOCHROME B
5′ss: 5′ splice sites
