# Supplementary material for: Functional intron-derived miRNAs and host-gene expression in plants
Source: Plant Methods. 2018 Sep 24;14:83. doi: 10.1186/s13007-018-0351-2 (PMC6151947; doi:10.1186/s13007-018-0351-2)
Supplement: Supplementary file 1 — Additional file 1: Figure S1. Nucleotide sequences of all ff-gLUC/aimiRNA constructs used in this study. gatatc: − EcoR V site; gaattc: EcoR I site; capital letter: exon sequences; small letters black: intron sequences; small letters blue: Id-amiRNA sequences based on miR319a. Specific sequences targeting TCP, LUC or PHYB are underlined. 5′intron splice sequences are boxed (ag: 3′intron splice site, gt: 5′intron splice site). Figure S2. RT-PCR products sequence from ffgLUCdelaimiR-319a transgenic plant. Figure S3. A similar transformation efficiency were confirmed by quantify the DsRED gene expression in transient assay samples. The quantification data is normalized against the N. benthamiana reference with UBI3 as internal control. Error bars represent standard error. Figure S4. Confirmation of mature aimiR-319a expression in stem-loop RT-PCR assay. Illustration of RT-PCR method for amplification of mature microRNA (A). Gel electrophoresis results from stem-loop RT (B). ffgLUC plant used as a positive control for endogenous mature miR319a. A mature microRNA specific forward primer and universal reverse primer were used for PCR amplification. Mature microRNA products were obtained using 25 cycling of RT-PCR and analysed on 4% agarose gel in 1xTAE. Predicted products were compared with 50 bp DNA ladder. Figure S5. PHYB silencing phenotype under constant cR. Table S1. Stable transformation of Arabidopsis WT or Arabidopsis line ff-gLUC-1 with the different expression constructs. NA not applicable, *very low LUC activity; **low LUC activity. Table S2. List of primers were used in this work. [file 13007_2018_351_MOESM1_ESM.pdf]

> Genomic Luciferase sequence (ff**gLUC**)

ATGGAAGACGCCAAAAACATAAAGAAAGGCCCGGCGCCATTCTATCCGCTAGAGGATGGAACCGCT
GGAGAGCAACTGCATAAGGCTATGAAGAGATACGCCCTGGTTCCTGGAACAATTGCTTTTACAGATG
CACATATCGAGGTGAACATTACgtaagtttctgcttctacctttgatatatatataataattatcattaattagtagtaatata
atatttcaaatatttttttcaaaataaaagaatgtagtatatagcaattgcttttctgtagtttataagtgtgtatattttaatttataactt
ttctaatatatgaccaaaatttgttgatgtgcagGTACGCGGAATACNNNN.......NNNNTAA

> Id-amiR-319a in intron of ffgLUC (ff**gLUC**$^{\text{aimiR-319a}}$)

ATGGAAGACGCCAAAAACATAAAGAAAGGCCCGGCGCCATTCTATCCGCTAGAGGATGGAACCGCT
GGAGAGCAACTGCATAAGGCTATGAAGAGATACGCCCTGGTTCCTGGAACAATTGCTTTTACAGATG
CACATATCGAGGTGAACATTACgtaagtttctgcttctacctttgatatatatataataattatcattaattagtagatatcag
agagcttccttgagtccattcacaggtcgtgatatgattcaattagcttccgactcattcatccaaataccgagtcgccaaaattcaaa
ctagactcgttaaatgaatgaatgatgcggtagacaaattggatcattgattctctttgattggactgaagggagctccctgaattcgt
aatataatatttcaaatatttttttcaaaataaaagaatgtagtatatagcaattgcttttctgtagtttataagtgtgtatattttaattt
ataacttttctaatatatgaccaaaatttgttgatgtgcagGTACGCGGAATACNNNN.......NNNNTAA

> Id-amiR-LUC combined ffgLUC intron sequence (as used ff**gLUC**$^{\text{aimiR-LUC}}$)

ATGGAAGACGCCAAAAACATAAAGAAAGGCCCGGCGCCATTCTATCCGCTAGAGGATGGAACCGCT
GGAGAGCAACTGCATAAGGCTATGAAGAGATACGCCCTGGTTCCTGGAACAATTGCTTTTACAGATG
CACATATCGAGGTGAACATTACgtaagtttctgcttctacctttgatatatatataataattatcattaattagtagatatcta
taactgcctgcctcagataaggtcgtgatatgattcaattagcttccgactcattcatccaaataccgagtcgccaaaattcaaactag
actcgttaaatgaatgaatgatgcggtagacaaattggatcattgattctctttaatctgacgcaggcagttctagaattcgtaatata
atatttcaaatatttttttcaaaataaaagaatgtagtatatagcaattgcttttctgtagtttataagtgtgtatattttaatttataactt
ttctaatatatgaccaaaatttgttgatgtgcagGTACGCGGAATACNNNN.......NNNNTAA

> Id-amiR-PHYB combined ffgLUC intron sequence (as used ff**gLUC**$^{\text{aimiR-PHYB}}$)

ATGGAAGACGCCAAAAACATAAAGAAAGGCCCGGCGCCATTCTATCCGCTAGAGGATGGAACCGCT
GGAGAGCAACTGCATAAGGCTATGAAGAGATACGCCCTGGTTCCTGGAACAATTGCTTTTACAGATG
CACATATCGAGGTGAACATTACgtaagtttctgcttctacctttgatatatatataataattatcattaattagtagatatcta
gctgtaaaccgtaaggctcaggtcgtgatatgattcaattagcttccgactcattcatccaaataccgagtcgccaaaattcaaacta
gactcgttaaatgaatgaatgatgcggtagacaaattggatcattgattctctttcagcctttcggtttacagttagaattcgtaatata
atatttcaaatattttttcaaaataaaagaatgtagtatatagcaattgcttttctgtagtttataagtgtgtatattttaatttataactt
ttctaatatatgaccaaaatttgttgatgtgcagGTACGCGGAATACNNNN.......NNNNTAA

> Id-amiR319a in intron ffgLUC with deletion of intron sequence (ff**gLUC**$_{del}^{aimiR-319a}$)

ATGGAAGACGCCAAAAACATAAAGAAAGGCCCGGCGCCATTCTATCCGCTAGAGGATGGAACCGCT
GGAGAGCAACTGCATAAGGCTATGAAGAGATACGCCCTGGTTCCTGGAACAATTGCTTTTACAGATG
CACATATCGAGGTGAACATTACgtaagtttctgatatcagagagcttccttgagtccattcacaggtcgtgatatgattcaa
ttagcttccgactcattcatccaaataccgagtcgccaaaattcaaactagactcgttaaatgaatgaatgatgcggtagacaaattg
gatcattgattctctttgattggactgaagggagctccctgaattcaacttttctaatatatgaccaaaatttgttgatgtgcagGTAC
GCGGAATACNNNN.......NNNNTAA

Figure S1. Nucleotide sequences of all ff-gLUC/aimiRNA constructs used in this study. gatatc: – EcoR V site; gaattc: EcoR I site; Capital letter: exon sequences; small letters black: intron sequences; small letters blue: Id-amiRNA sequences based on miR319a. Specific sequences targeting TCP, LUC or PHYB are underlined. 5'intron splice sequences are boxed (ag: 3'intron splice site, gt: 5'intron splice site)

> CDS Luciferase sequence (**correct spliced**)

ATGGAAGACGCCAAAAACATAAAGAAAGGCCCGGCGCCATTCTATCCGCTAGAGGATGGAACCGCT
GGAGAGCAACTGCATAAGGCTATGAAGAGATACGCCCTGGTTCCTGGAACAATTGCTTTTACAGATG
CACATATCGAGGTGAACATTACGTACGCGGAATACTTCGAAATGTCCGTTCGGTTGGCAGAAGCTAT
GAAACGATATGGGCTGAATACAAATCACAGAATCGTCGTATGCAGTGAAAACTCTCTTCAATTCTTTA
TGCCGGTGTTGGGCGCGTTATTTATCGGAGTTGCAGTTGCGCCCGCGAACGACATTTATAATGAACG
TGAATTGCTCAACAGNNNNNNNNNNNNNNNNNNNNNNNNNNNNNNNNNNNNNNNNNNNNNNNN
NNNNNNNNNNNNNNNNNNNNNNNNNNNNNNNNNNNNNNN

> shorter CDS Luciferase sequence (**3'splice site shifted**)

ATGGAAGACGCCAAAAACATAAAGAAAGGCCCGGCGCCATTCTATCCGCTAGAGGATGGAACCGCT
GGAGAGCAACTGCATAAGGCTATGAAGAGATACGCCCTGGTTCCTGGAACAATTGCTTTTACAGATG
CACATATCGAGGTGAACATTACAGCTATGAAACGATATGGGCTGAATACAAATCACAGAATCGTCGT
ATGCAGTGAAAACTCTCTTCAATTCTTTATGCCGGTGTTGGGCGCGTTATTTATCGGAGTTGCAGTTG
CGCCCGCGAACGACATTTATAATGAACGTGAATTGCTCAACAGNNNNNNNNNNNNNNNNNNNNN
NNNNNNNNNNNNNNNNNNNNNNNNNNNNNNNN

> shorter CDS Luciferase sequence (**5'and 3'splice site shifted**)

ATGGAAGACGCCAAAAACATAAAGAAAGGCCCGGCGCCATTCTATCCGCTAGAGGATGGAACCGCT
GGAGAGCAACTGCATAAGGCTATGAAGAGATACGCCCTGGTTCCTGGAACAATTGCTTTTACAGATG
CACATATCGAGGAGCTATGAAACGATATGGGCTGAATACAAATCACAGAATCGTCGTATGCAGTGAA
AACTCTCTTCAATTCTTTATGCCGGTGTTGGGCGCGTTATTTATCGGAGTTGCAGTTGCGCCCGCGAA
CGACATTTATAATGAACGTGAATTGCTCAACAGNNNNNNNNNNNNNNNNNNNNNNNNNNNNNN
NNNNNNNNNNNNNNNNNNNNNNNNNNNNNNNNNNN

Figure S2. RT-PCR products sequence from ffgLUC$_{del}^{aimiR-319a}$ transgenic plant.

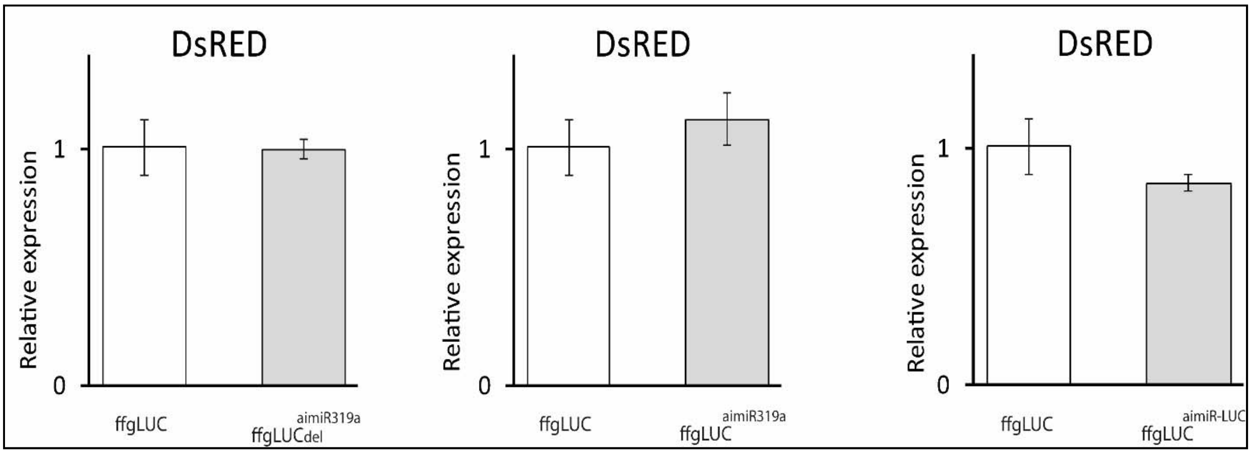

Figure S3. A similar transformation efficiency were confirmed by quantify the DsRED gene expression in transient assay samples. The quantification data is normalized against the *N.benthamiana* reference with UBI3 as internal control. Error bars represent standard error.

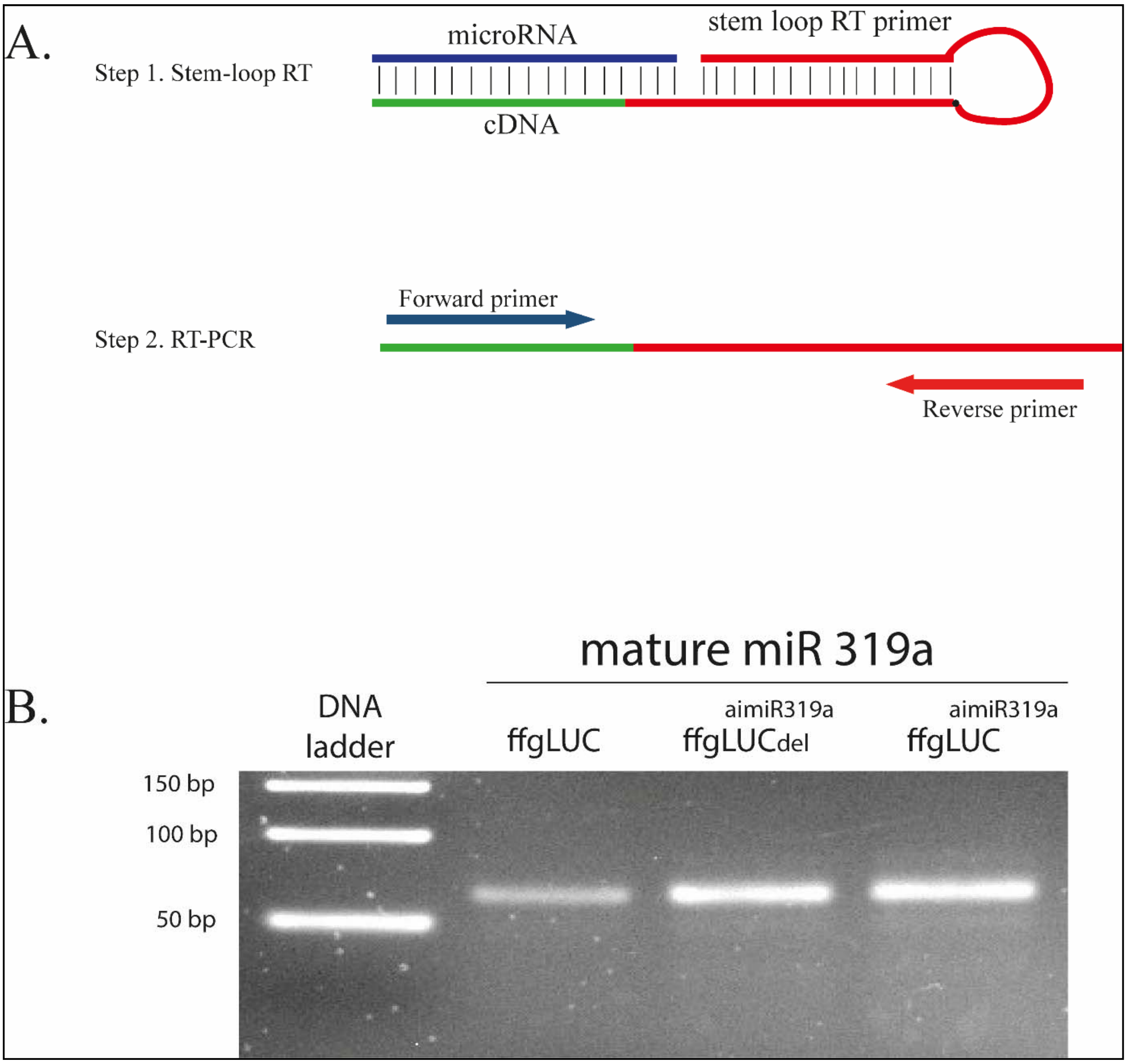

Figure S4. Confirmation of mature aimiR-319a expression in stem-loop RT-PCR assay. Illustration of RT-PCR method for amplification of mature microRNA **(A)**. Gel electrophoresis results from stem-loop RT **(B)**. ffgLUC plant used as a positive control for endogenous mature miR319a. A mature microRNA specific forward primer and universal reverse primer were used for PCR amplification. Mature microRNA products were obtained using 25 cycling of RT-PCR and analysed on 4% agarose gel in 1xTAE. Predicted products were compared with 50bp DNA ladder.

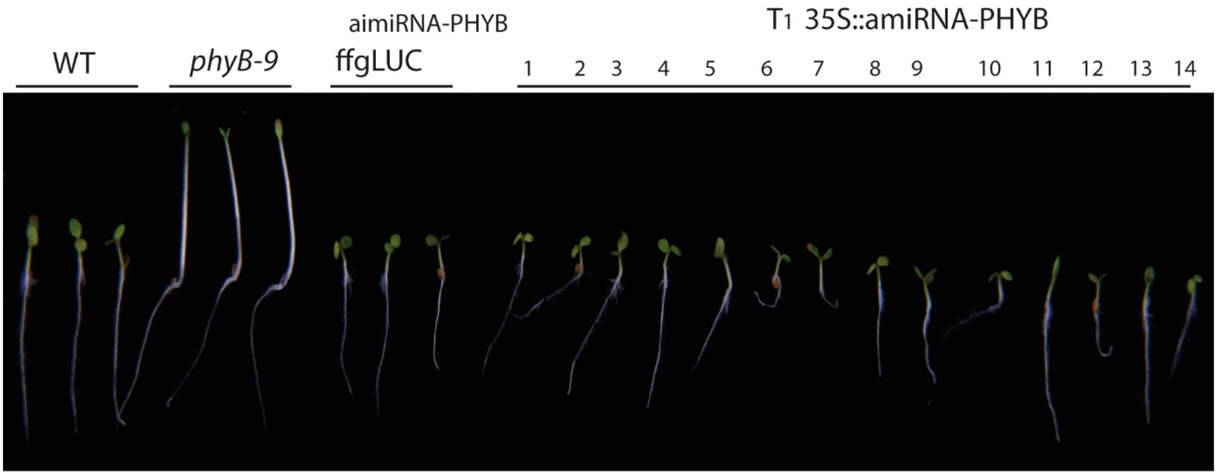

Figure S5. PHYB silencing phenotype under constant cR.

**Transformation in *A.thaliana***	**Positive seeds**	**#transformants tested**	**#LUC expression**	**#TCP silencing/ leaf phenotype**	**#LUC silencing**	**#PHYB silencing**
35S::ffgLUC	NA	NA	NA	NA	NA	NA
35S::ffgLUC$_{del}^{aimiR\text{-}319a}$	>25	17	17*	17	NA	NA
35S::ffgLUC$^{aimiR\text{-}319a}$	>25	19	19	17 (2 dead)	NA	NA
35S::ffgLUC$^{aimiR\text{-}LUC}$	>20	20	20**	NA	20	NA
35S::ffgLUC$^{aimiR\text{-}PHYB}$	>12	10	10	NA	NA	
35S::amiR-LUC/35S::ffcLUC	>19	19	19**	NA	19	NA

Table S1. Stable transformation of Arabidopsis WT or Arabidopsis line ff-gLUC-1 with the different expression constructs. NA: not applicable, * very low LUC activity; ** low LUC activity

PRIMER NAME	SEQUENCE	USED FOR	REF
Luc-F-NcoI	agcaaaccATGGAAGACGCCAAAAAC	cloning of full LUC gene	In study
Luc-R-NotI	agcaaagcggccgcTTACAATTTGGACTTTCCGCCCTT	cloning of full LUC gene	In study
Luc-intron-R-EcoR V	aaagatatcTACTAATTAATGATAATTATT	cloning of first exon+ intron LUC	In study
Luc-intron-F-EcoR I	aaagaattcGTAATATAATATTTCAAATATTTTTTTCAAAATAA	cloning of intron + second exon LUC	In study
miR319a_F-EcoR V	aaagatatcAGAGAGCTTCCTTGAGTCCATTCAC	cloning of miRNA319a	[1]
miR319a_R-EcoRI	tttgaattcAGGGAGCTCCCTTCAGTCCAATC	cloning of miRNA319a	[1]
AmiRphyB-F-EcoR V	aaagatatcTAGCTGTAAACCGTAAGGCTCAGGTCGTGATATGATTCA	cloning of artificial miR-PHYB	In study
AmiRphyB-R-EcoR I	aaagaattcTAACTGTAAACCGAAAGGCTGAAAGAGAATCAATGATCCA	cloning of artificial miR-PHYB	In study
AmiRluc-F-EcoR V	aaagatatcTATAACTGCCTGCCTCAGATAAGGTCGTGATATGATTCA	cloning of artificial miR-LUC	In study
AmiRluc-R-EcoR I	aaagaattcTAGAACTGCCTGCGTCAGATTAAAGAGAATCAATGATCCA	cloning of artificial miR-LUC	In study
Luc splice 2_R-EcoR V	aaagatatcAGAAACTTACGTAATGTTCACCTCG	cloning of first exon + intron splice LUC	In study
Luc splice 2_F-EcoR I	aaagaattcAACTTTTCTAATATATGACCAAAATTTGTT	cloning of intron splice + second exon LUC	In study
*Ath*PHYB_F	CGTTGGGTGTTGCTCCTAGT	qPCR in *A.thaliana* (At2g18790)	In study
*Ath*PHYB_R	GATACCCCGCATCGCCTAAA	qPCR in *A.thaliana* (At2g18790)	In study
*Ath*TCP2_F	AACGGCGGAGCATTCAATCTT	qPCR in *A.thaliana* (At4 g18390)	[2]
*Ath*TCP2_R	GCCTTTACCCTTATGTTCTGA	qPCR in *A.thaliana* (At4 g18390)	[2]
*Ath*TCP3_F	CATCCAGTTTATAGCCAAA	qPCR in *A.thaliana* (At1 g53230)	[2]
*Ath*TCP3_R	ATGGCGAGAATCGGATGAA	qPCR in *A.thaliana* (At1 g53230)	[2]
*Ath*TCP4_F	CCTTCAACGACGTCGTTTCAGCCAG	qPCR in *A.thaliana* (At3 g15030)	[2]
*Ath*TCP4_R	GTGAACCGGTGGAGGAAGGTGATG	qPCR in *A.thaliana* (At3 g15030)	[2]
*Nb*TCP4_F	CTGCATCTGCTGCAAACATT	qPCR in *N.benthamiana* (Niben101Scf01002g02011.1)	In study
*Nb*TCP4_R	AACCCATTGGGAAAAAGGAC	qPCR in *N.benthamiana* (Niben101Scf01002g02011.1)	In study
*Ath*Actin F	GGTAACATTGTGCTCAGTGGTGG	qPCR in *A.thaliana*	In study
*Ath*Actin R	AACGACCTTAATCTTCATGCTGC	qPCR in *A.thaliana*	In study
*Nb*Ubi3_F	GCCGACTACAACATCCAGAAGG	qPCR in *N.benthamiana*	In study
*Nb*Ubi3_R	TGCAACACAGCGAGCTTAACC	qPCR in *N.benthamiana*	In study
DsRED F	GAAGCTGAAAGACGGTGGTC	qPCR in *N.benthamiana*	In study
DsRED R	CGTCCCTCGGTTCTTTCATA	qPCR in *N.benthamiana*	In study
LUC-RT-F	CGAGGTGAACATTACGTAAGTTTC	RT-PCR	In study
LUC-RT-R	GTATTCCGCGTACCTGCAC	RT-PCR	In study
MIR-319A RT	GTCGTATCCAGTGCAGGGTCCGAGGTATTCGCACTGGATACGACAGGGAG	RT-PCR	In study
MIR-319A F	CGGCGGTTGGACTGAAGGGAG	RT-PCR	In study
AMIRPHYB RT	GTCGTATCCAGTGCAGGGTCCGAGGTATTCGCACTGGATACGACTAACTG	RT-PCR	In study
AMIRPHYB F	CGGCGGCAGCCTTTCGGTTTA	RT-PCR	In study
UNIVERSAL REVERSE	GTGCAGGGTCCGAGGT	RT-PCR	[3]

Table-S2. List of primers were used in this work.

Reference

1. Liang G, He H, Li Y, Yu D: **A new strategy for construction of artificial miRNA vectors in Arabidopsis.** *Planta* 2012, **235:**1421-1429.
2. Nag A, King S, Jack T: **miR319a targeting of TCP4 is critical for petal growth and development in Arabidopsis.** *Proc Natl Acad Sci U S A* 2009, **106:**22534-22539.
3. Varkonyi-Gasic E, Wu R, Wood M, Walton EF, Hellens RP: **Protocol: a highly sensitive RT-PCR method for detection and quantification of microRNAs.** *Plant Methods* 2007, **3:**12-12.
